# Psychometric Properties of the SAS, BAI, and S-AI in Chinese University Students

**DOI:** 10.3389/fpsyg.2019.00093

**Published:** 2019-01-31

**Authors:** Zhanyu Pang, Dongbo Tu, Yan Cai

**Affiliations:** School of Psychology, Jiangxi Normal University, Nanchang, China

**Keywords:** psychometric properties, item response theory, SAS, BAI, S-AI

## Abstract

Three widely-used self-report anxiety scales, including the Self-Rating Anxiety Scale (SAS), the Beck Anxiety Inventory (BAI), and the State Anxiety Inventory (S-AI), were used to simultaneously compare the psychometric properties via an item response theory (IRT) model with Chinese university students as the sample. Although these scales were probably to measure the same underlying construct, namely, anxiety, their psychometric properties were different. Results showed that the BAI’s measurement error was fewer than that of the other scales, with their anxiety severity ranging approximately from the 0.8 standard deviations below the mean to 3 standard deviations above the mean, while the S-AI’s measurement error was fewer than that of the other degrees of anxiety. The S-AI provided more information than the other scales when the student’s scale was less than approximately 0.8 standard deviations below the mean of anxiety severity. In general, the BAI showed better, for it provided more information than the other scales at the broadest range of anxiety severity. The SAS provided less information than the other scales at all anxiety severity range. In conclusion, BAI shows good psychometric quality. Finally, the three instruments were combined on a common scale by using IRT model and a conversion table was provided so as to achieve the transformation of each scale score.

## Introduction

Anxiety is an unpleasant state of fear and uneasiness. It is an irritating emotion caused by excessive worry about the life safety of relatives or themselves, their future and fate, etc. People with serious anxiety can also cause emotional and emotional disorders. Anxiety is a prevalent emotional disorder that interferes with psychosocial functioning (Balestrieri et al., 2010). Thus, it is not surprising that most anxiety assessment tools have been developed into clinical settings. Anxiety disorders have become a serious public health problem in China. As a study (Phillips et al., 2009) published in the international authoritative medical journal *The Lancet* shows, based on the calculations of epidemiological data of the four provinces, including Shandong, Zhejiang, Qinghai, and Ningxia, the number of patients with anxiety disorders in China is as high as 57 million. But 90% of the patients are untreated. The diagnosis rate and treatment rate of anxiety disorder in China are low. At the beginning, it did not attract enough attention of people, who would consider whether they were suffering from anxiety disorder only when they had received repeated medical and surgical inspections and ineffective treatments. Therefore, many people with anxiety disorder were not diagnosed and treated in time. Among individuals with anxiety disorder, only 8% had ever sought professional help and 5% had ever seen professional mental health doctors. Anxiety disorder, if has not been correctly identified and treated, will not only bring spiritual pain to people, but also bring a heavy social and economic burden to the country. Anxiety is further divided into state anxiety and trait anxiety. State anxiety refers to the temporary passive state of a person, which exists immediately and has a certain level of intensity. Trait anxiety refers to stable individual differences in a person’s relatively long-lasting personality traits. There are two ways to measure anxiety: self-reported and evaluations by others.

With the rapid development of society and the increase of competitive pressure, anxiety has become a common psychological problem for college students that affects their healthy growth. In China, some epidemiological surveys (Demirci et al., 2015) indicate that anxiety and depression are the main health problems among college students that have the greatest impact on their daily lives.

Adopting anxiety self-report scales to assess the degree of anxiety is a commonly used measuring method for college students. Therefore, selecting an effective anxiety scale is crucial for assessing anxiety symptoms and conducting timely intervention.

Assessing anxiety in Chinese university students by using self-report inventories is very prevalent in the past few decades. Plenty of different self-report scales have been adopted in previous researches, including the Interaction Anxiousness Scale (Leary and Kowalski, 1993), the Hamilton Anxiety Scale (Maier et al., 1988), the Self-Rating Anxiety Scale (SAS), the Beck Anxiety Inventory (BAI), the State Anxiety Inventory (S-AI), and so on. Despite some differences concerning item numbers, symptom coverage, and so on, each scale measures the same underlying construct, that is, anxiety. Selecting one inventory over another is usually guided by psychometric functioning, historical preference, and training for a specific measure. In an era of heightened attention to empirically based assessments, using data to guide measurement selection is especially important (Hunsley and Mash, 2008).

Most self-report measurement of psychological constructs have been evaluated by relying on classical test theory (CTT), which lays emphasis on internal consistency, test–retest stability, as well as construct validity (Hunsley and Mash, 2008). However, CTT methods fail to offer direct guidance in terms of accurately assessing anxiety symptomatology at various points of the anxiety severity range. This goal may be realized through methods of the item response theory (IRT). As the basis of modern psychometric techniques, IRT methods can provide estimations of individual latent trait (e.g., anxiety severity) and item characteristics. Item- and test-information functions can be generated by integrating parameters estimation in IRT models, which can graphically describe and most precisely evaluate the regions of the latent trait continuum.

In IRT, item- and test-information functions are evaluated on the same latent trait instrument, so they can be comparable across inventories (Embretson and Reise, 2004). Therefore, simultaneously comparing multiple instruments on a single and common metric can be achieved with the help of IRT analyses. The IRT methods provide estimations about latent traits on the position where each item or inventory lies the most information (Olino et al., 2012).

There are a growing number of studies that have employed CTT and IRT approaches to assess self-report anxiety scales. These studies hold several different objectives. First, some have examined the psychometric properties of a single instrument (Fydrich et al., 1992; Steer et al., 1993; Vigneau and Cormier, 2008; Lindsay and Michie, 2010). These investigations provide information about a specific instrument that is independent of other measures. Several studies were conducted to examine the factor structure of the anxiety scales (Shek, 1988; Hewitt and Norton, 1993; Olatunji et al., 2006), but the findings were inconclusive and in some cases may be two to four factors. Second, some studies have linked with various scale scores (Beck and Steer, 1991; de Ayala et al., 2005; Covic et al., 2012; Le Blanc et al., 2014), and developed a short form (Li and Lopez, 2007). However, only a few studies have inspected the functioning of multiple instruments (Quintão et al., 2013). The investigation examined the validity study of the BAI by the Rasch rating scale model (RSM), and compared the state–trait anxiety and Zung SAS by test the functionality of the response category systems. These previous investigations cannot reflect situations, concerning whether diverse instruments can provide valid and precise information across the same or different trait levels or not. Further, there is evidence that in the actual application, using different scales, the assessment results vary widely, and the demarcation of the scale is often more difficult to unity. These suggest that further investigations on the psychometric properties of self-report anxiety scales are necessary. In addition, though the scales are frequently adopted in researches and practice, there is no study that compared multiple anxiety scales shifted in Chinese samples via IRT approaches at the same time.

In this study, we have investigated and compared the psychometric properties of these self-report anxiety scales (SAS, BAI, and S-AI) by using an IRT model. These scales (SAS, BAI, and S-AI) has become a common practice to assess anxiety among university students in China over the past several decades (Wenli, 1995; Dong et al., 2011; Gao et al., 2012; Zhang et al., 2014). The study is expected to give guidance for determining which scale to use in a given application context or a given study design. Moreover, which scale can provide greater information in a lager range of anxiety is also checked. At last, a conversion table was provided so as to achieve the transformation of each scale scores.

## Materials and Methods

### Participants

A final total of 1,109 participants were recruited from Jiangxi Normal university, Zhengzhou university, and Luoyang Normal university in China ranging from freshman to senior students (male: 421, female: 688). Their age is ranged from 16 to 25, and the mean age of them was 20.30 years (*SD* = 1.47).

The current study was conducted in conformity to the recommendations of psychometrics studies on mental health at the Research Center of Mental Health, Jiangxi Normal University, and approved by the Research Center of Mental Health, Jiangxi Normal University, and the Ethics Committee of Jiangxi Normal University. The written informed consent was obtained from all participants in accordance with the Declaration of Helsinki.

### Measures

Three widely used self-report instruments to access anxiety were employed here, which were the SAS, BAI, and S-AI. They have both demonstrated high levels of internal consistency and test–retest stability (Barnes et al., 2002; Ramirez and Lukenbill, 2008; Chapman et al., 2009; Cao and Liu, 2015). Responses for the SAS are frequency-based, possessing four response options that range from “rarely,” “none of the time,” to “most all of the time.” Responses for the BAI and S-AI are severity-based, and each of them contains four options of different severity degrees. The SAS, BAI, and S-AI assessed symptoms by combining with participants’ experience in the past week. All participants completed the Chinese version questionnaire of the three scales.

#### Self-Rating Anxiety Scale (SAS; Zung, 1971)

The SAS is a well-accepted instrument for adults and adolescents to make self-report measurement of anxiety in both clinical and research settings, which includes four groups of manifestations: motor, autonomic, cognitive, and central nervous system symptoms. The SAS containing multiple choices, altogether 20 items, is a self-report inventory that measures the anxiety severity among adults and adolescents. The items reflect common symptoms of anxiety—for example, “I feel tense and anxious than usual and my hands are numb or tingling.” Of the 20 items, five items were negatively worded. According to Ramirez and Lukenbill (2008), the SAS had great internal consistency reliability coefficient of 0.80. The Chinese version of SAS (Lindsay and Michie, 2010) for people with a mental handicap, the findings indicated that the SAS had strong internal consistency (α = 0.92). In the present study, the Chinese version of SAS had a Cronbach’s alpha of 0.78 and a split-half reliability of 0.75. The total scores were multiplied by 1.25, and then the integers were taken as the standard score. When SAS standard score ≥ 45, it showed a tendency of anxiety.

#### Beck Anxiety Inventory (BAI; Beck et al., 1988)

The BAI scale is composed of 21 items, which are descriptive statements of anxiety symptoms, and participants have to evaluate themselves according to their own condition, based on scoring in two components: cognitive and somatic. The BAI is a four-point and 21-item Likert scale, which has four options ranging from option 1 (no symptom) to option 4 (severe symptoms can only be tolerated). The items reflect common symptoms of anxiety—for example, heart palpitations or heart rate increase. Of the 21 items, no item was negatively worded. In previous studies (Fydrich et al., 1992), The BAI proved highly internal consistent (Cronbach’s alpha = 0.94) and acceptably reliable over an average time lapse of 11 days (*r* = 0.67). In the study of the Brazilian version of BAI had excellent reliability, with a Cronbach α of 0.91 for psychiatric samples, 0.86 for clinical samples, and 0.86 for non-clinical samples. In the present study, the Chinese BAI version the scale had a Cronbach’s alpha of 0.95 and a split-half reliability of 0.92. The total scores were multiplied by 1.19, and then the integers were taken as the standard scores. Higher standard scores indicate severer anxiety symptoms. Participants were regarded as slightly anxiety with the BAI standard scores (>45).

#### State Anxiety Inventory (S-AI; Spielberger et al., 1970)

The S-AI quantitatively assesses the participants’ anxiety severity and diagnostic status within the past week. State anxiety can be defined as fear, nervousness, discomfort, etc. The S-AI scale had 20 statements refers more to how a person is feeling at the time of a perceived threat. Among all the items, half of them were describing negative emotion and the rest were describing active emotion. The S-AI is a four-point and 20-item Likert scale that has four options ranging from option 1 to 4. Assessment toward each item: 1—completely without, 2—some, 3—middle degree, 4—very obvious. These items assisted to survey the frequency of such symptoms, like “I feel upset or terrible in daily life” or “I feel anxious or nervous.” Of the 20 items, 10 items were negatively worded. In previous studies, Cronbach’s alpha has been found to range from 0.86 to 0.95 for the subscale S-AI (Spielberger et al., 1970), whose scores have adequate test-retest reliability in multiple time intervals (Barnes et al., 2002). In the present study, the Chinese version of S-AI had a Cronbach’s alpha of 0.90 and a split-half reliability of 0.87. The total score could be obtained by adding the score of 21 items together, and when S-AI total score (≥45), it showed a tendency of anxiety.

### Procedure

The informed consent was made for all participants. If participants agree to take part in this study, they will be asked to read the introduction of a questionnaire and to finish the questionnaire in accordance with their true feeling of the latest week, including the same day. Participants were assured that all of their information were anonymously and would be strictly kept confidential. The information collected is only for research purposes. All the participants completed the Chinese version three questionnaire scales in a fix order simultaneously. Under the uniform instruction, participants were required to finish the questionnaires independently and hand in them on the spot. If the participants want to know their own results, they can email us.

### Analysis Process

We first assessed internal consistency for each scale by calculated Cronbach’s alpha. Then the unidimensionality of three scales was checked using Exploratory Factor Analysis (EFA) and Confirmatory Factor Analysis (CFA). Finally, IRT models were employed to fit these scales.

#### EFA and CFA

Although IRT has lots of merits, its models are built on a quantity of assumptions. One important assumption of them is that the underlying trait being tested is unidimensional. Both the EFA and the CFA were implemented here, in order to ensure that the unidimensional assumption was met or to study whether the constructs assessed by the items of each scale represent the same construct, First, this sample was randomly divided into two groups of nearly identical size: a development sample (*n*1 = 554) and a validation sample (*n*2 = 555). Then, we conducted EFA of all the items together of three scales with the development sample. Finally, the CFA was followed with the validation sample.

In EFA, we focus on the ratio of the first to the second eigenvalue, and the variance explanation rate of the first eigenvalues. Within CFA, we supposed that each scale had one factor that was correlated with the other two factors.

#### IRT Analyses

Several widely used polytomous IRT models were applied to fit the data, and then the most suitable one of them was selected for the subsequent analysis. The polytmous IRT models used here including the Graded Response Model (GRM; Samejima, 1969), the Generalized Rating Scale Model (GRSM), the Generalized Partial Credit Model (GPCM; Muraki, 1997), and the RSM (Andrich, 1978).

The common-used, test-level, and model-fit indices were employed here to choose the models that fit IRT best, which included −2log-likelihood (−2LL; Spiegelhalter et al., 1998), Akaike’s information criterion (AIC; Akaike, 1974), and Bayesian information criterion (BIC; Schwarz, 1978).

With the estimated parameters, we assessed the standard error of measurement (SEM), the category response curve, test information, and relative efficiency of this study. Moreover, combining with estimated parameters of the GRM, we examined how scale scores would be transferred to theta values, and we calculated each scale’s scores in light of estimated values.

The software R (Version3.3.2^1^) and the R packages ltm (Version 1.11; Rizopoulos, 2006), and mirt (Version1.24; Chalmers, 2012) were employed to estimate item parameters and model selection. Other statistical analyses were conducted by using Mplus (Version 7.0; Muthén and Muthén, 2012) and SPSS (Version 23.0; George, 2016).

## Results

### Descriptive Statistics

In the sample, the mean (and standard deviation) of the three scales scores were as follows, SAS: 44.56 (8.64), BAI: 36.22 (11.80), and S-AI: 41.79 (9.45). According to the cutoff scores presented in previous studies of Chinese samples (Li and Qianming, 1995; Shek, 1988; Zheng et al., 2002), 647 and 498 participants were addressed as no anxiety symptoms on the SAS (<45) and the S-AI (<40), respectively, while on the BAI, 877 participants appear slight anxiety symptoms (<45).

### EFA and CFA

The internal consistency coefficient (i.e., Cronbach’s alpha coefficient) of item pool of three scales were investigated to explore whether the constructs of item pool of three scales met the unidimensional hypothesis of IRT, and then the EFA and CFA were conducted with the development sample (*n*1 = 554) and the validation sample (*n*2 = 555), respectively.

Cronbach’s alpha was 0.784 for the SAS, 0.950 for the BAI, and 0.895 for the S-AI. Cronbach’s alpha coefficient of item pool of three scales was 0.951. The high internal consistency indicated that item pool of three scales had one main structure—anxiety.

The EFA results showed that the ratio of the first to the second eigenvalue reached 3.161 and the first eigenvalues explained 28.96% variance of all. According to previous studies (Zickar and Broadfoot, 2009; Slocum-Gori and Zumbo, 2011), in which the data were deemed to be unidimensional when the ratio of the first eigenvalue to the second eigenvalue was over 3 or the first eigenvalues explained over 20% variance of all, the item pool of three scales overall was likely to have one-factor structure (anxiety), which was consistent with previous studies with Chinese university sample. After the EFA, the item pool was then submitted to CFA, a high-order factor model with one second-order factor (anxiety) and three first-order factors (one first-order factor for each scale) and use the validation sample. The Tucker–Lewis index (TLI) is a good fit; the comparative fit index (CFI) and the root-mean-square error of approximation (RMSEA) were 0.84 and 0.054, respectively. Although the CFI was lower than.90, the RMSEA is within the acceptable range; *x*^2^(1728, *N* = 1099) = 7372.354, *P* < 0.001. In addition, after the correlations among the three scales’ total scores were calculated, the results showed that there were strong positive correlations among each scale (*r* = 0.54–0.66).

Strict unidimensionality is never achieved in practice; however, results of internal consistency coefficient, EFA, and CFA indicated that the total item pool of three scales as a whole was likely to meet the unidimensionality assumption in Chinese university sample, for the purposes of calibrating three scales by using IRT models.

### IRT Model Selection

In order to select a particular IRT model, we employed the GRM, GPCM, RSM, and GRSM to calibrate parameters and then evaluated the model-data fit indexes including −2LL, AIC and BIC, which are documented in Table 1.

**Table 1:** Test-level model fit for four polytomous scored IRT models.

Model	−2LL	AIC	BIC
GRM	114,102.7	114,590.7	115,813.4
GPCM	114,551.4	115,039.4	116,262.1
RSM	121,146.8	121,274.8	121,595.5
GRSM	116,305.7	116,553.7	117,175.1

*−2LL = −2log-likelihood; AIC = Akaike information criterion; BIC = Bayesian information criterion; GRM = Graded Response Model; GPCM = Generalized Partial Credit Model; RSM = Rating Scale Model; GRSM = Generalized Rating Scale Model.*

From Table 1, the GRM was the best fit model in that the −2LL, AIC, and BIC were all the lowest in all models. The GRM is suitable for the analysis of this kind of Likert-type data. The GRM is more consistent with the actual situation, and then the GRM model was selected to estimate item parameters.

### IRT Analysis

Given that the GRM fitted the data best, the GRM was finally used to analyze the item responses of the SAS, BAI, and S-AI, and to estimate item parameters to compare measurement equivalence and psychometric properties.

First, we calculated the severity and discrimination parameters of all items that belong to the three measures. For the SAS, it ranged from 0.16 to 1.59, and severity parameters ranking first, second, and third ranged from −3.26 to 1.11, −0.47 to 4.24, and 3.32 to 6.44, respectively. For the BAI items, it ranged from 1.03 to 2.85, and severity parameters ranking first, second, and third ranged from −0.83 to 1.16, 1.29 to 2.27, and 2.56 to 4.45, respectively. For the S-AI items, it ranged from 0.41 to 1.65, and severity parameters ranking first, second, and third ranged from −3.97 to 0.58, −0.20 to 2.25, and 2.28 to 6.44. These item parameters make a great difference to item information curves. As the height of the curve showed, curves with higher information along the θ scale obtain better measurement precision. Due to it that all the individual item information functions constitute the test information function, the more information each item can contribute, the more information the whole measurement will offer.

Second, we drew category response curves for each item based on the estimated parameters. From the category response curves, we observed the choices for some items of the SAS were not able to function well (Items 5, 9, 13, 17, and 19), these items were all negatively worded. For Items 5 (I think everything is fine and nothing unfortunate happens) and 9 (I feel calm and easy to sit quietly), the higher the degree of anxiety, the less likely the Category 4 (representing the highest anxiety severity) being selected. For Items 17 (My hand is often dry and warm) and 19 (I am easy to fall asleep and sleep well all night long), the Category 2 or 3 had less probability to be selected within the range of anxiety severity. This may result in weaker discrimination. In addition, the options for some items of the S-AI were improbable to work well (Items 1, 2, 5, 8, 10, 11, 15, 16, 19, and 20), these items were negatively worded. For Item 5 (I feel comfortable), the higher the degree of anxiety, the less likely the Category 4 being selected. For other items, Category 2 or 3 did not have the highest probabilities of being selected over the range of anxiety severity, which may also result in weaker differentiation. However, the items of BAI, which have different shapes and locations on category response curves, and students with different anxiety degrees would have different aggregate scores, then the BAI items are not negatively worded. Therefore, the BAI could be used to discriminate various degrees of anxiety among Chinese university students.

**FIGURE 1:**
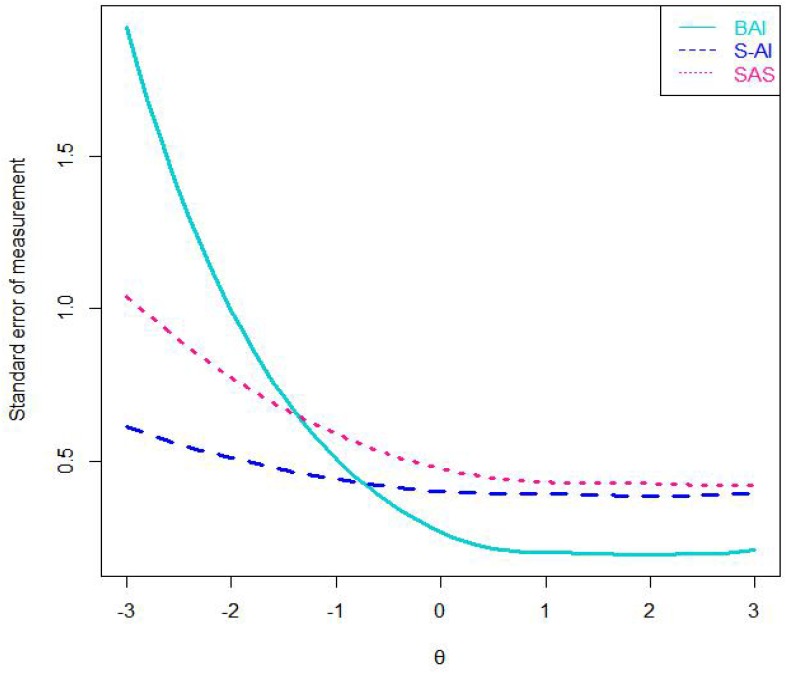
The standard error of measurement of three scales. Note: SAS = the Self-Rating Anxiety Scale; BAI = the Beck Anxiety Inventory; S-AI = the State Anxiety Inventory.

Third, we calculated the SEM for each scale. SEM is the key indicator of the quality of the inventories, and the smaller of the SEM, the higher reliability and the greater the amount of information will be. An important feature of IRT models is that the SEM is described as a function conditional on values of θ. Another advantage of IRT is that individuals’ θ estimates are independent of the number of items or the specific items used for testing (Quintão et al., 2013). As shown in Figure 1, among the three scales, the S-AI’s measurement error is smaller than the others at the anxiety severity range that goes approximately from −3 standard deviations to the 0.8 standard deviations below the mean, while at the other anxiety severity range, the BAI’s measurement error is smaller than the other scales. The BAI performed better in the whole process, because it provided less measurement error than the other scales within the maximum range of anxiety severity.

**FIGURE 2:**
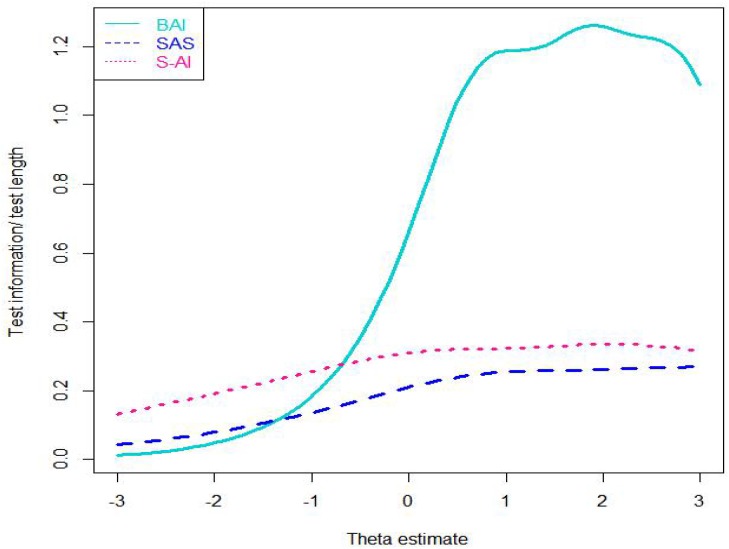
Average item information curves of three scales. Note: SAS = the Self-Rating Anxiety Scale; BAI = the Beck Anxiety Inventory; S-AI = the State Anxiety Inventory.

Fourth, we calculated the total test information of the three scales. The value of the total test information is the inverse of a squared standard error’s anxiety estimate value, and it can indicate measurement precision for anxiety (Umegaki and Todo, 2016). Given that the amount of test information increases with the length of a scale extends, we divided the total test information value by each scale’s length value, and then we could examine average item information value of the scale (AII; see Figure 2), which indicated the information per item contained at each nodes along the θ scale. The more information there was, the higher precision and reliability of the measurement would be. As indicated in Figure 2, of the three scales, the S-AI’s AII best assessed anxiety symptomatology at the range approximately from −3 standard deviations to 0.8 standard deviations below the mean. Almost in other ranges of θ, the BAI’s AII was the highest among the three scales. Conversely, the SAS’s AII was lower than that of the other scales. These results conformed to the fluctuation of the category response curves, which showed that the BAI was good for assessing information of various degrees of anxiety severity. However, these scales failed to assess information very well at any anxiety levels.

**FIGURE 3:**
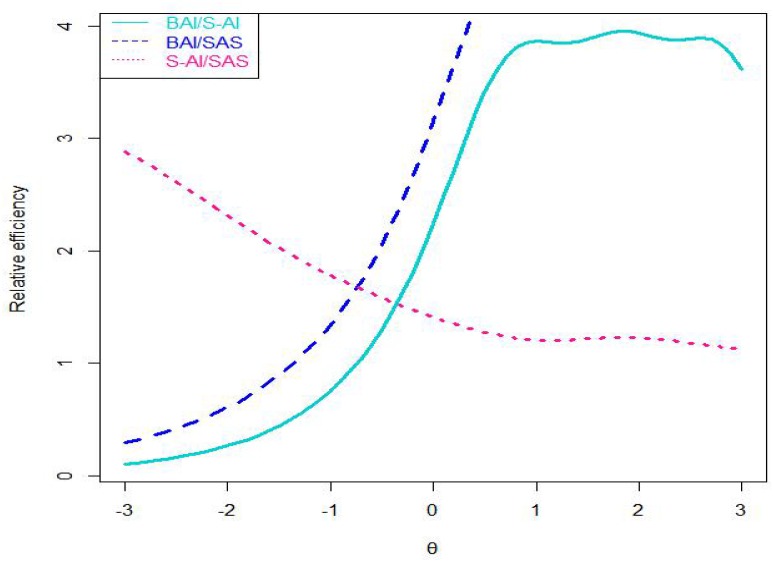
Curves of relative efficiency of three scales. Note: SAS = the Self-Rating Anxiety Scale; BAI = the Beck Anxiety Inventory; S-AI = the State Anxiety Inventory.

Next, we illustrated relative efficiency curves of the three scales (see Figure 3). Given that the BAI have 21 items and S-AI have 20 items, we use value of 1.1 (i.e., $\frac{21}{20}$ ≈ 1.1) to compare the scales. The relative efficiency of the BAI compared to that of the S-AI is likely to be greater than 1.1 at the range from approximately 0.8 standard deviations below the mean to 3.0 standard deviations above the mean of anxiety severity index, and conversely it is less than 1.1 at the range lower than 0.8 standard deviations below the mean. This means that, when comparing the BAI scale with the S-AI scale, the BAI can better discriminate students with anxiety degree around or above the average level, while the S-AI do well in discriminating students with anxiety degree below the average level. Furthermore, the relative efficiency of the BAI compared to that of the SAS is higher than 1.1 at the range from approximately 1.2 standard deviations below the mean of anxiety severity index to 3.0 standard deviations above it, while the SAS provides more information in other degrees of anxiety. In addition, the relative efficiency of the S-AI compared to that of the SAS was greater than 1 at all the anxiety levels. The SAS and S-AI are of the same length, which means that when comparing the SAS with the S-AI, the S-AI provides more information for the students at the all different anxiety levels.

Finally, we calculated the expected scores of the three scales with various degrees of anxiety by relying on estimated item parameters, the expected scores was calculated by transferring theta values, and then we created a conversion table (see Table 2).

**Table 2:** Conversion table of three scales based on expected scores.

θ Scale	SAS	BAI	S-AI	θ Scale	SAS	BAI	S-AI
−3	29.98	25.02	24.81	0.1	43.09	32.03	42.22
−2.9	30.17	25.02	25.17	0.2	43.89	33.14	43.05
−2.8	30.36	25.03	25.54	0.3	44.71	34.41	43.88
−2.7	30.56	25.04	25.91	0.4	45.57	35.85	44.71
−2.6	30.77	25.05	26.31	0.5	46.44	37.46	45.53
−2.5	30.99	25.06	26.71	0.6	47.33	39.21	46.35
−2.4	31.21	25.07	27.12	0.7	48.23	41.08	47.15
−2.3	31.45	25.09	27.55	0.8	49.15	42.99	47.93
−2.2	31.7	25.11	27.99	0.9	50.07	44.9	48.71
−2.1	31.97	25.14	28.43	1	50.99	46.76	49.48
−2	32.24	25.17	28.89	1.1	51.91	48.56	50.25
−1.9	32.54	25.21	29.36	1.2	52.83	50.3	51.03
−1.8	32.85	25.26	29.84	1.3	53.73	52.01	51.81
−1.7	33.18	25.31	30.34	1.4	54.63	53.74	52.61
−1.6	33.53	25.38	30.84	1.5	55.52	55.55	53.44
−1.5	33.89	25.46	31.36	1.6	56.41	57.47	54.29
−1.4	34.28	25.56	31.89	1.7	57.31	59.53	55.16
−1.3	34.7	25.67	32.44	1.8	58.21	61.68	56.07
−1.2	35.13	25.81	33.00	1.9	59.12	63.89	56.99
−1.1	35.59	25.97	33.58	2	60.04	66.07	57.93
−1	36.08	26.17	34.17	2.1	60.99	68.16	58.88
−0.9	36.59	26.39	34.79	2.2	61.95	70.14	59.83
−0.8	37.12	26.66	35.43	2.3	62.94	72.02	60.78
−0.7	37.68	26.98	36.10	2.4	63.95	73.86	61.71
−0.6	38.26	27.34	36.78	2.5	64.97	75.7	62.63
−0.5	38.87	27.77	37.50	2.6	66	77.59	63.53
−0.4	39.5	28.26	38.23	2.7	67.05	79.53	64.4
−0.3	40.17	28.82	38.99	2.8	68.09	81.49	65.26
−0.2	40.85	29.47	39.78	2.9	69.13	83.4	66.1
−0.1	41.57	30.21	40.58	3	70.16	85.21	66.93
0	42.32	31.06	41.39				

*SAS = the Self-Rating Anxiety Scale; BAI = the Beck Anxiety Inventory; S-AI = the State Anxiety Inventory.*

The test equivalent means that the scores on different tests that measure the same psychological quality are converted into unit systems to achieve a process that can be compared with each other. Here, we provide more details of GRM, which is widely used in mental health and Likert-type data. Analogous to two-parameter logistic model, GRM has one discrimination parameter and a group of severity parameters where each severity parameter is a between-category “threshold.”

Using the GRM, we calculated the individual’s probability of responding in a specific response category used Equations (1) and (2).

(1) $$P_{\mathrm{jt}}^{*}=\frac{1}{1+\exp[-{\mathrm{Da}}_{j}(\theta_{i}-b_{\mathrm{jt}})]}$$

(2) $$P_{\mathrm{jt}}=P_{\mathrm{jt}}^{*}-P_{j,t+1}^{*}$$

where θ_*i*_ is the ability parameter for examinee *i*; *D* is a scale factor, generally taken as 1. 7; *a_j_* is the discrimination parameter for item*j*; *b_jt_* is the *t*th threshold parameter for item *j*, which satisfies *b*_*j*1_ < *b*_*j*2_ < ⋯ < *b_jmf_j__*; *mf_j_* represents the maximum score of item *j*. $P_{\mathrm{jt}}^{*}$ expresses the cumulative likelihood of examinee *i*, getting at least a score *t* on item*j*, and *P_jt_*(θ_*i*_) is the likelihood of examinee *i*, responding to item *j* in a particular category score *t*. In addition, it assumes that $P_{j0}^{*}$ = 1 and $P_{j,{\mathrm{mf}}_{j}+1}^{*}=0$.

The expected scores can be derived by multiplying each probability of responding in specific response category with the corresponding score, and then adding them together.

## Discussion

The study analyzed the psychometric properties of the three frequently used self-report anxiety scales in Chinese university students: the SAS, BAI, and S-AI. Alpha coefficients, strong positive correlations among each scale, and the results of the EFA and CFA show that item pool of three scales had high internal consistency and as a whole measured the same construct—anxiety. Although the scales can be used to measure the same underlying construct—anxiety, the results based on IRT indicated that each scale appears to have different psychometric properties. By observing category response curves, SEM, and relative efficiency, we got that at the range approximately from the mean −3 to 0.8 standard deviations below the mean of anxiety severity, the S-AI provides more information than the other two scales, while more information is provided by the BAI for the other degrees of anxiety. The SAS performed worse on all the anxiety severity. Therefore, when measuring anxiety severity among no anxiety symptoms Chinese university students, the S-AI seems to be the best choice, while the BAI would be a good one when recruiting those with elevated levels of anxiety symptoms.

The conversion of the three scale scores (see Table 2) enables conversion of one scale can be shifted into another one. This conversion table can be useful for future study and application when different scale scores need to be switched. The SAS adopts a cutoff score of 45 to discriminate anxiety patients from no anxiety symptoms patients (Chen and Yang, 2002) and the S-AI adopts 40 (Spielberger et al., 1970), the SAS’s cutoff score is easy to identify Chinese university students with anxiety more severe than approximately 0.35 standard deviations above the mean, while the S-AI’s cutoff score to identify those with anxiety more severe than approximately 0.15 standard deviations below the mean of anxiety severity. On the other hand, the BAI adopts cutoff scores of 45 (Kabacoff et al., 1997), the BAI’s cutoff score is likely to identify Chinese university students with anxiety more severe than approximately 0.95 standard deviations above the mean. At the cut-off of the SAS, BAI, and S-AI, the approximately average item information is 0.2, 1.2, and 0.3, respectively. In conclusion, at these cut-off of these scales, we can find that BAI provided more information than the other scales. From the conversion table, we can also know the scores of three different anxiety scales corresponding to the same θ level. According to the conversion table of three scales, we can find the standard of the S-AI scale is more rigorous, because more college students with anxiety symptoms are marked.

## Limitations and Future Directions

The study is significant in that it directly compared the psychometric properties of commonly used self-report anxiety scales in a large Chinese university student sample which uses an IRT model. Guidance will be given for determining which scale to use in a given application context or a given study design. Compared with the other two measures, the BAI assesses anxiety within a broader range of severity with greater precision, so it is suitable to be used at the situations where high levels of anxiety symptom tends to happen—for example, experimental researches and clinical diagnosis. BAI’s brevity and simplicity make it an ideal instrument for use as a pretest for presence of anxiety disorder, which is consistent with the scientific literature (Quintão et al., 2013). The S-AI is likely to be applied to prevention studies, where below-normal to medium levels of anxiety symptom are expected. The largest contribution of this research was to give guidance for psychological health screening of college students. Taking the psychological health screening of college students in Chinese universities, for example, first, we can use the S-AI scale to find students with anxiety symptoms, and then let these students complete the BAI scale. Based on their scores on the BAI scale, we can judge their degree of anxiety and use different assistance plans accordingly. By combining two S-AI and BAI scales, we can assess anxiety symptoms more effectively and conduct timely intervention.

Although the three scales are seemed as unidimensionality in the present study, the Chinese SAS often reflects two or more factors when applied in university students (Olatunji et al., 2006; Wang, 2011). Additionally, two or more factors are reported by previous studies for the S-AI (Shek, 1988; Barnes et al., 2002). Thus, the SAS and S-AI should be tested further based on multidimensional models. Of course, there is also an interesting study to use higher order IRT model to fit these scales. A higher order IRT model is a multidimensional IRT model which includes some sub-dimensions and a higher order dimension loading on sub-dimensions. Another limitation of this study was the fact that it was not used a clinical sample, being suggested for future studies the use of clinical samples, with medical or psychiatric disorders. Future researches should contribute to targeting a larger community sample, for example, teenagers and adults. In addition, inclusion of other frequently used self-report anxiety scales, such as the HAMA and the Interaction Anxiousness Scale. Development of a scale that assesses information very well at any anxiety level is also a future direction.

## Author Contributions

DT and YC selected the topic and made some modifications of the paper. ZP collected the data and wrote the manuscript.

## Conflict of Interest Statement

The authors declare that the research was conducted in the absence of any commercial or financial relationships that could be construed as a potential conflict of interest.

## Footnotes

1 http://www.R-project.org/
